# Medical Miscellanies

**Published:** 1816-08

**Authors:** 


### PART IV.

### MEDICAL MISCELLANIES.

### MORBID ANATOMY.

The great work of Morgagni, "*de Sedibus et Causis Morborum,*" is not in the hands of one in one hundred of the medical practitioners of Great Britain; nor has it been probably perused by one in five hundred. The reasons are—its vast extent—(three quarto volumes) its epistolary circumlocution—and its antiquated physiology. Could it be divested of the two last, the first objection would vanish of course; and it would then certainly present a most interesting and instructive picture to the thinking and unthinking part of the profession.

The Editors, wishing to stimulate by all possible means to the study of morbid anatomy, believe that they cannot do it more effectually than by keeping the attention of their readers constantly excited to the subject, by laying before them a condensed, yet faithful miniature of the great work above-mentioned, interspersing occasional observations of their own, as circumstances may require.

If, after repeated examinations of this great work, and now that Time has strewed his silvery mementos over our heads, we still think the hours well spent in reperusing and transcribing its most prominent features; it cannot be any insult even to the more dignified members of the profession, to offer them an epitome of what they have sometimes read, and often had occasion to refer to.

Forsan et hæc olim meminisse juvabit.

To the lower, but by far the more numerous orders of medical society, whose avocations or patience will not permit them to explore so vast a collection, the manner and mode of publication here adopted, cannot but be highly useful and agreeable; as they will be, as it were, insensibly led through a short and easy journey, where they would otherwise expect a long and a toilsome march.

How far the interwoven remarks of the Editor, who has imposed on himself this task, may conduce to the utility of the plan, must be left to time, and to the judgment of the public to decide. They will be carefully distinguished from the original text, that the reader may have an opportunity of censuring or approving them as he may think fit.

For the style, the Editor himself is responsible; as the condensed *matter* of each passage in the original or translation is to be conveyed entirely in the language of the Editor.

For the sake of reference, the letter of Morgagni, in which each article is found, will be annexed to the extract.

Valeat quantum valere debet.

### SEDES et CAUSÆ MORBORUM;

(Selected and abridged from MORGAGNI.)

WITH COMMENTARIES.

### DE DOLORE CAPITIS.

*Lett. I.*—Headache attends many diseases whose seats are in other parts than the head. Idiopathic headache is seldom mortal—few histories of it are introduced here—and those shew it only as the precursor of other disorders, or a troublesome attendant on them.

*Art. I.*—A youth, ætat. 12, whose brother and sister had died of phthisis, suffered pulmonic inflammation of the left side. Twelve months afterwards, he was seized with pain over the orbits—in the eyes, with viscid lachrymal discharge. Second day, delirium—vomiting—convulsions—lethargy—and at length death.

*Sectio Cadaveris. Abdominal* viscera sound. In the stomach, a greenish liquid—bladder turgid with urine—gall bladder with bile. *Thorax.* Tubercle as large as a walnut in the right lung, containing pus. Left lung adherent to the pleura costalis. In the pericardium, two ounces of water. In the right ventricle of the heart, a small polypous concretion.

*Head.* Dura mater tinged of a cineritious colour along the blood-vessels. On tearing this covering from the crista galli, some coloured serum burst forth. An ounce of limpid serum came from the origin of the optic nerves. Brain otherwise sound.

Much unintelligible pathological reasoning follows, which deserves no remark. That inflammation, or at least turgesence of the brain, succeeded by effusion, caused the death of the patient, few English physicians will doubt. No treatment is detailed.

*Art. II.*—A man, ætat. 40, had been afflicted for many years with periodical pain in the right hypochondrium; frequently attended with vomiting, and even ileus. To these were added almost constant headache, and lachrymal discharge from the eyes. After a fit of intoxication, he was attacked with the usual hypochondriac pain and vomiting, which were removed by an empirical unction applied to the abdomen. Immediately afterwards, he was seized with vehement headache, both internally and externally. The nostrum was then applied to the head, which increased the pain. Delirium and convulsions followed. These ceasing about an hour before death, difficult respiration — foaming at the mouth—strong full pulse, and other apoplectic symptoms closed the scene.

*Post mortem appearances.* Face pale, limbs contracted—pericranium, about the sinciput, thickened by effusion—serum betwixt the pia mater and brain—water in the ventricles. In the abdomen, some effused serum—an indurated liver.

The doctrine of sympathetic influence between the liver and the brain has lately gained ground. That in the foregoing case, a morbid determination to the brain was the consequence of hepatic obstruction and induration, may be fairly inferred. What the Italian treatment was, cannot now be ascertained; but among the enlightened part of the profession here, the cerebral determination would have been met by vigorous local depletions, not from the head alone, but from the region of the liver; while powerful drastic purgatives roused the action of the abdominal viscera, and determined as much as possible of the circulation through the descending aorta and its various outlets.

As a specimen of Morgagni's physiological reasonings may be *amusing*, we shall here insert one. "The diseased liver must necessarily secrete a vitiated bile, which when poured out into the duodenum must cause pains in those parts, and ultimately produce vomiting, ileus, &c. The causes of the disease (i. e. vitiated bile) being imprudently repelled by the unction, became inherent in the nearest membrane without the cranium in the form of a jelly; and within the cranium also, there irritating the pia mater, &c. and causing the pain, delirium, convulsions, and at length apoplexy."

However unable we may be to explain the nature of metastasis, *a change of determination* in the vascular system points to a better mode of treatment, than the *humoral* doctrines above adduced.

*Art. III.* A young married woman, the daughter of an epileptic mother, being heated and fatigued by a long journey, was attacked by violent headache, and acute fever, without delirium; but she appeared reservedly silent. With these symptoms she died, in three or four days. As she suckled, and yet menstruated, bleeding was long deferred; but getting daily worse, while the vascular action was very strong, eight ounces of blood were abstracted from the foot, which quickly and firmly coagulated. She died immediately afterwards.

*Dissection.* The head only could be procured. The inside of the skull had a reddish brown appearance. A yellowish kind of matter, of a thick consistence, and inodorous, was diffused between the dura and pia mater. It appeared to the physicians present to be purulent.

Remarks. No abscess or other source of pus being discovered, much speculation is entertained on the fluid above mentioned. Morgagni balances in opinion whether it was pus translated from another part of the body, or a kind of exudation from the vessels of the meninges themselves. Although accumulations of different kinds of fluids often disappear from one part, while a similar fluid is deposited in another, yet it is difficult to say, whether it is a translation of matter, or a metastasis of action, if the phrase be allowed. The latter appears more consonant with the physiological and pathological ideas of the present time.

The doctrine of *revulsion* was here acted on, after much unnecesſary fear about lactation and menstruation — circumstances that would be now disregarded when an important organ was oppressed.

The practice of drawing blood from a part remote from the seat of disease, as in the above instance, by way of revulsion, seems the result of an erroneous theory. Blood drawn from the foot can produce no more derivation from the head than if it were drawn from the arm, or any part of the trunk; and can act only as depleting the whole sanguiferous system. If bleeding produced a derivation of blood *from another* part *to* the part from whence the blood is taken, then topical blood-letting would be manifestly prejudicial in local inflammation, which is not the case.

A derivation however may be effected, by the application of such s[illegible]ances to a *remote* part, as may cause inflammation there. Thus blisters will excite inflammation—a determination of blood to the part where applied, and ultimately *effusion* there, which cures the inflammation itself, while the new determination of blood reduces that of another part. It is no argument against this, that blisters to the chest relieve affections of the chest. The new determination to the *surface*, with effusion under the cuticle, takes off the determination of blood to the pleura costalis or pulmonalis within. The same applies to affections of the head or any other part where the inflammation is deep-seated; but no one would apply a blister to an external inflammation—as erysipelas.

### Epistle II. and III. Sanguineous Apoplexy.

The following passage in Celsus appears to apply to catalepsy rather than apoplexy. "Attonitos quoque *raro* videmus, quorum et corpus, et mens stupet. Fit interdum ictu fulminis, interdum morbo, hunc ἀποπληξίαν Græci appellant." A little further on he says, At resolutio nervorum frequens ubique morbus est. Sed interdum tota corpora, interdum partes infestat. Veteres authores illud *apoplexiam*, hoc *paralysin* nominarunt."

So frightful and dangerous a disease as apoplexy, must have excited great attention from the days of Hippocrates to the present time; and much discussion has occurred respecting its nature and causes. Its great divisions, however, have been into sanguineous and serous.

Case I. *Cardinal Sanvitalis*, of moderate stature, full muscular habit, and florid complexion, studious, arthritic; had been, some years before, attacked with an irritation of the fauces, accompanied by ineffectual efforts to spit; to which were added, at intervals, convulsive motions of the hands and feet. At the age of 45, having resided for a few months in a mountainous country, exposed to the southernly winds, which had previously disagreed with him; and being also in a state of mental disquietude, he was attacked with vertigo, which being removed, was succeeded by depression of spirits and drowsiness. In twenty days, the vertigo returned, accompanied by vomiting; both of which disappearing, a violent headache succeeded. The following day, and at the same hour of the vertiginous attack, he was seized with *hemiplegia* of the left side, and lay as though he were in a profound sleep. The respiration was natural—pulse frequent, large and vehement. Stimuli applied to the right side and nostrils slightly roused him, so as to make signs, and even articulate some words. After venæsection the irritability became greater, especially on the sixth day of the apoplexy, when, by Valsalva's order, the right jugular vein was opened. In four hours after the operation, his internal senses were awakened, and his speech for an hour was restored. The same change took place at the same hour on the following day, but was more evident, and of longer duration. This was the last glimpse of hope, for from this time he gradually declined, with convulsive motions of the right side—intermissions of the pulse, &c. till death, which occurred on the tenth day.

*Sectio Cadaveris.* Every viscus in the thorax and abdomen was found in a natural state. The brain was flaccid —In the left ventricle a little serum; in the right, more than two ounces of coagulated blood. The plexus chorodes was here torn through, and the parietes of the ventricle corroded into the form of a deep ulcer.

Remarks. Of all diseases, the premonitory symptoms of apoplexy require the most vigilant attention. In the case before us, determination of blood to the head was early evinced by the affection of the fauces, and the convulsive motions of the upper and lower extremities; more obviously still by the repeated vertigoes, and *unequivocally* at last, by the violent pain in the head. These were the times for not only lessening the general vascular plethora, by venæsection, purgatives and abstinence, but of attempting a derivation of blood from the sensorium, by establishing a drain in some part of the body. We are at this moment attending a gentleman, 80 years of age, who for six or eight months, has had several of the abovementioned precursory symptoms of apoplexy; but as he enjoyed uninterrupted health from infancy up to octogenerean age, he perversely refused to take any medicine, or suffer any local evacuation from the head. The consequence was, that eight days ago, he was suddenly seized with hemiplegia of the left side, and the general symptoms of apoplexy. From the first moment, the pulse was tumultuous and intermitting; the face slightly suffused—the eyes and mouth pouring forth tears and saliva — the face drawn slightly to one side. A large blister was applied between the shoulders—brisk cathartics exhibited, and stimulating pediluvia applied to the lower extremities. The next day, he regained the use of the left arm and leg — became sensible of what was said to him, and could articulate pretty clearly. Evacuations from the bowels, and from the blistered surface were kept up, and some faint hopes were entertained of at least a partial recovery; but these were blasted in two days more, by a total loss of power in the side originally affected, with insensibility, stertorous snoring, and coma. He is now (the 9th day) moribund.

Morgagni makes an ingenious remark here, while endeavouring to account for the utility of bleeding from the jugular vein in apoplectic attacks. He observes, that as the external jugular vein principally returns the blood carried to the head by the branches of the *external* carotid artery, it follows, that when the blood is evacuated from the vein by an external orifice, more than usual of the blood ascending by the common carotid trunk is distributed to the external branches of the artery to supply this waste, and consequently less is driven up through the *internal* carotid to the brain. The same reasoning will probably explain the utility of temporal arteriotomy in fevers of the warmer regions, where the head is so much oppressed with blood; and also the beneficial effects of local abstractions of blood, by cupping, leeches, &c. from the *exterior* of the head, when the interior is affected.

Case 2. *From Valsalva also.* A man, 60 years of age, of a sanguineous temperament, and of a good habit of body, fell by accident, in walking, and struck his head violently against the ground. Being slightly stupid—his forehead bruised— blood gushing out of his nostrils, with paralysis of the left arm, which was senseless as well as motionless, he was brought into the Hospital *de Santa Maria*, at Bologna. His face was flushed—respiration laborious pulse hard, and moderately quick. On the fourth day he was speechless—on the fifth he died. No treatment is detailed.

*Sectio Cadaveris.* Thoracic and abdominal viscera sound. Upon opening the head no fracture of the skull was found. The dura mater shewed a slight mark of contusion, which did not reach to the pia mater. In the right ventricle of the brain, two ounces of coagulated blood. The corpus striatum, with part of the plexus choroides so much eroded, that scarcely a vestige remained

REMARKS. This case is introduced by Morgagni, in order to state a difference of opinion that prevailed between Valsalva and himself respecting it. Valsalva attributed the effusion of blood in the ventricle to the fall—Morgagni the fall to the effusion of blood. We are fully of Morgagni's opinion; though we think it not improbable that the hæmorrhagic effusion into the ventricle was increased, or its effects exasperated, by the contusion on the head.

CASE 3. *From Valsalva.* A woman, 70 years of age, had for many months, felt a gradual decay of memory, and also of vision, when objects were placed in a certain position. When walking, she scarcely raised her feet from the ground. She had been seized, about twelve months previously, with a disorder in the head, from which she speedily recovered; but now she fell down suddenly, while eating, with hemiplegia of the left side, and paralysis of the right arm. Her respiration was perfectly natural; as was also the colour of her face, which, in her, was pale; nor did any convulsions appear. Her head fell, like that of a corpse, into any position to which it was thrown. She exhibited no sense of understanding or feeling, except a slight degree of motion when an incision was made into the jugular vein. She died in nine hours. Both ventricles of the brain were filled with fluid blood; the right, much eroded, as well about the external margin of the corpus striatum as of the thalamus nervi optici. The plexus choroides could scarcely be observed. The other parts were sound.

REMARKS. This case shews us how difficult it is to form a diagnosis between sanguineous and serous apoplexy! The septuagenary age of the woman—the pallid countenance—and the perfectly natural state of the respiration, were little indicative of both ventricles being filled with blood; and even in these *phlebotomising* days, would pretty certainly doom the man to an imputation of madness who took the lancet in his hand on such an occasion.

Yet does the acute Morgagni sanction venæsection in these cases, and asserts that he saved the life of a relation of his, a nun, of 80 years, more than once, by bleeding and other evacuations, when she was attacked with apoplexy, regardless of the age, and only looking to the symptoms.

Morgagni here adverts to some histories and dissections which would lead us to believe that clots of effused blood are sometimes absorbed, leaving traces of their former existence in the substance of the brain. To shew that the body sometimes suffers on the same side as where the brain is affected, the following short history is given.

Case 4. An old man of 70, fell down suddenly on the ground, having lost the power of feeling and moving on the left side, the right being agitated by convulsions. His face was red. He expired in less than 24 hours. His head being opened, coagulated blood was found between the right posterior lobe of the brain and the dura mater, and a kind of concreted serum betwixt the sanguiferous vessels of the pia mater, which being cut through, some water oozed out.

Remarks. Here the convulsions of the right side of the body are attributed to the pressure on the right side of the brain; yet the hemiplegia was on the left, or opposite side, as usual.

Case 5. A man, 58 years of age, of a good natural constitution, but intemperate in the use of tobacco, fell down suddenly, while buckling his shoes, with the entire loss of speech and motion. His face was first pale; then grew yellow as in jaundice, and afterwards pale again, while some saliva flowed from the mouth. He died in a quarter of an hour. In the cranium, a good quantity of coagulated blood was found under the pia mater on the anterior convexity of the brain, particularly on the right side. In both ventricles also, some blood was effused. The yellow suffusion on the face, and its sudden disappearance, are here very unaccountable.

Case 6. A stout young man, 22 years of age, used such violent exercise in running after the chariot of his master, as to throw him into a profuse perspiration, though the snow was falling. He went to his usual occupations in the evening without changing his clothes; but on getting out of bed next morning, he fell down headlong three times, with loss of sense. On being lifted up, he complained of deep-seated pain in the head; especially in the occiput. This was succeeded by fever, with an unusual lassitude, and pain all over the body. The following day, he was purged with the pil. galeni. On the third day, he was bled, but in vain; for the disorder increasing, he became lethargic. On the fifth day he was cupped, aud scarified between the shoulders. On the eighth day, he had an apoplectic fit for an hour; after which the pain in the occiput was much exasperated, and extended to the shoulders and spine. On the ninth day, he was bled from the other arm, which was followed by a remission of the symptoms; but the apoplectic paroxysm returning, he died.

*Sectio Cadaveris.* Where the medulla makes its exit from the cranium, some grumous blood was found, which had flowed from the lacerated trunk of the internal carotid artery. The ventricles of the brain contained a great quantity of saltish water; the lateral ventricles contained also a portion of coagulated blood. Throughout the crura of the medulla oblongata, many little prominent bodies were seen, which, except that they were pellucid, resembled grains of millet seed.

( To be continued. )

---

*An Account of a monstrous Production, preserved in the Royal Museum of Anatomy at Berlin.*

THIS most remarkable monstrosity, exhibiting a head without body or arms, together with the following account of it, was sent to the Royal Anatomical Museum, by Dr. Elfes, of Neuss.

On the 18th of October, 1815, Catharine Hannen, 21 years of age, was delivered, at a quarter past five o'clock in the morning, of a boy; at six o'clock of another; and soon afterwards of a head destitute of a body.

The first born, a fine and more complete looking infant, whose fonticuli were small, and who exhibited few marks of præmaturity, died on the 20th, at eleven o'clock in the morning, after a restless night, some slight spasms, and a previous loss of a few drops of blood from the nose. He had not taken the breast, and sucked only some sugar water, sweet fennel water, and syrup of rhubarb, which had carried off the excrements. He weighed four pounds and two ounces common weight, and measured fourteen inches.

The second, with an elderly countenance, and all the characteristics of præmaturity, broad and long fonticuli, and a much leaner body than the first, had also not taken the breast, but more sugar water, sweet fennel water, and syrup of rhubarb, than the other, and had likewise had evacuation. On the same day, towards noon, he became restless, was taken with slight spasms, lost a couple of tea-spoonfuls of blood from the nose, and died in the afternoon at five o'clock. He weighed two pounds ten ounces and three-quarters, common weight, and measured near eleven inches. Nothing unusual was to be seen in either of them.

The solitary head weighs four ounces and three-quarters; has on the great protuberance a skin two inches long, with two veins as substitutes for the umbilical cord, by which it probably adhered to the coats of the ovum, and anastomosed with the cord of one of the two placentæ.

The midwife assisting at the labour, being an ignorant woman, and having no notion of the theoretical and practical consequence of this phænomenon, never tried whether the head possessed life when first born, and did not even apprize a medical man of the fact; and the author, hearing of it in the afternoon by mere accident, got possession of the head and the two placentæ, but was not able to procure the two children.

The mother had been married to a labourer, John Peter Hannen, since the 25th of September, 1814, and had her courses up to the 5th or 6th of March. She could not remember having quickened. During the last six weeks of her pregnancy, the milk frequently flowed from her breasts; but nothing particular had happened during that period. Both the man and his wife are healthy and well formed, and neither of them belongs to the class of debilitated habits. The husband is rather of a phlegmatic habit, but the woman more of a sanguine temper. There is no hereditary bodily defect in either of their families. She had a good time, and went abroad soon afterwards. Her mother once had twins. The midwife, notwithstanding all the questions put to her, could tell the author no more of the whole proceeding of parturition, than that the head had been enclosed in a bladder and joined to one of the placentæ; that she had opened the bladder and extracted the head.

No similar case can with certainty be proved. There is, however, an account to be found in Lycosthenes, and copied from him by Delrio Irenæus Licetus and others, which, throwing aside the fabulous investiture of the narrator, certainly appears to belong to this sort. The following are Licetus's own words (De monstris, Ex recensione Ger. Blasii Amstelodami 1665, 4to. p. 56): "Augustæ Vindelicorum mulier primo peperit caput humanum membranis involutum, mox, bipedem serpentem, cui lucii piscis caput, corpus ranæ, pedes et cauda lacertæ, tertio, porcillum."

The latter most likely were monstrous children, which, ad genium seculi, were taken for a biped serpent and a pig. *Communicated by Dr. Von Embden.*

---

" *Medical Statistics.*—Abstract of the Bills of Mortality, drawn up by the twelve municipalities of Paris, for the year 1815.—

| | | |
|---|---|---|
| Number of deaths in 1815 | - - - - - - - - - | 21,549 |
| ——————— 1814 | - - - - - - - - - | 27,778 |
| Diminution in - - 1815 | - - - - - - - - - | 6,229 |

| | | | |
|---|---|---|---|
| The deaths, in 1815, consist of | the male sex | 10,866 | 21,549 |
| | females - | 10,683 | |

In this number are included 175 suicides, and 279 bodies deposited at the dead-house; viz.

| | | |
|---|---|---|
| Males, 218; females, 61 | - - - - - - - | 279 |

Out of this number, there died at their own habitations,

| | | |
|---|---|---|
| Males, 7,225; females, 7,235 | - - - - - | 14,460 |

There died at the civil hospitals,

| | | |
|---|---|---|
| Males, 3,641; females, 3,448 | - - - - | 7,089 |

Dead of the small-pox, in 1815,

| | | |
|---|---|---|
| Males, 131; females, 59 | - - - - - - | 190 |
| The number of deaths from small-pox, in 1814, was | - | 534 |
| Diminution in 1815 | - - - - - - - - | 344 |

The deaths by suicide consist of
Males, 133; females, 42 - - - - - - 175

The number of births, in 1815, was, of
Males, 10,435; females, 9,747 - - - - 20,182

The number of deaths, therefore, exceeded the births by 1,367

The most remarkable diseases, on account of the number of persons destroyed by them, are the following

| | | | |
|---|---|---|---|
| Putrid or asthenic fevers - - - - | males<br>females | 678<br>660 | 1338 |
| Malignant fevers - - - - - - - | males<br>females | 361<br>388 | 749 |
| Anomalous fevers - - - - - - - | males<br>females | 265<br>260 | 525 |
| Inflammations of the skin - - - - | males<br>females | 348<br>336 | 684 |
| ——— of the mucous membranes | males<br>females | 1100<br>1150 | 2250 |
| ——— of the serous membranes | males<br>females | 115<br>164 | 279 |
| ——— of the cellular membrane and parenchymatous organs | males<br>females | 1071<br>1536 | 2607 |
| Comatose affections - - - - - - | males<br>females | 452<br>475 | 927 |
| Spasmodic affections - - - - - | males<br>females | 623<br>574 | 1197 |
| Local nervous affections - - - - | males<br>females | 530<br>550 | 1080 |
| General organic lesions - - - - | males<br>females | 2292<br>2370 | 4662 |
| Particular organic lesions - - - - | males<br>females | 661<br>788 | 1449 |
| Gangrenous inflammations - - - | males<br>females | 57<br>121 | 178 |

Women dead in Child-birth.

| | | | |
|---|---|---|---|
| From | 15 to 20 | years old | 4 |
| —— | 20 — 25 | ——— | 11 |
| —— | 25 — 30 | ——— | 12 |
| —— | 30 — 35 | ——— | 12 |
| —— | 35 — 40 | ——— | 11 |
| —— | 40 — 45 | ——— | 7 |
| | | Total | 57 |

Among the deceased, in consequence of general organic lesions, are an old man of 103 years; and a woman of 108."

"Recapitulation of the two sexes."

| Age | Males | Females | Total |
|---|---|---|---|
| "From 1 day to 3 months old | 3163 | 2029 | 5192 |
| — 3 months to 6 — | 191 | 173 | 364 |
| — 6 — to 1 year | 268 | 319 | 587 |
| — 1 year to 2 years | 503 | 528 | 1031 |
| — 2 years — 3 — | 296 | 306 | 602 |
| — 3 — — 4 — | 184 | 194 | 378 |
| — 4 — — 5 — | 164 | 168 | 332 |
| — 5 — — 6 — | 88 | 111 | 199 |
| — 6 — — 7 — | 91 | 102 | 193 |
| — 7 — — 8 — | 60 | 55 | 115 |
| — 8 — — 9 — | 40 | 58 | 98 |
| — 9 — — 10 — | 51 | 47 | 98 |
| — 10 — — 15 — | 182 | 182 | 364 |
| — 15 — — 20 — | 314 | 266 | 580 |
| — 20 — — 25 — | 331 | 447 | 778 |
| — 25 — — 30 — | 312 | 396 | 708 |
| — 30 — — 35 — | 268 | 388 | 656 |
| — 35 — — 40 — | 303 | 396 | 699 |
| — 40 — — 45 — | 261 | 417 | 678 |
| — 45 — — 50 — | 385 | 480 | 865 |
| — 50 — — 55 — | 410 | 412 | 822 |
| — 55 — — 60 — | 463 | 436 | 899 |
| — 60 — — 65 — | 593 | 548 | 1141 |
| — 65 — — 70 — | 490 | 546 | 1036 |
| — 70 — — 75 — | 529 | 539 | 1068 |
| — 75 — — 80 — | 380 | 477 | 857 |
| — 80 — — 85 — | 226 | 377 | 603 |
| — 85 — — 90 — | 84 | 169 | 253 |
| — 90 — — 95 — | 17 | 51 | 68 |
| — 95 — — 100 — | 1 | 5 | 6 |

"OBSERVATIONS.

"Although the number of deaths, in 1815, seems less considerable than in 1814, since it gives a diminution of 6229 persons, the relative mortality has yet been increased; because, in 1814, the foreign armies alone furnished 2569 deaths in the military hospitals, besides a considerable number in private houses; and because many people from the country had flocked to the suburbs of Paris. In 1815, the ordinary population was reduced one-fifth or more: consequently, the proportionate mortality is more considerable.

"Some diseases have been less fatal in 1815 than in the preceding year. Thus, typhus, which caused 414 deaths in 1814, produced but 115 in 1815; gastric fevers, 229 in 1814, but 152 in 1815; mucous fevers, 225 in 1814, only 113 in 1815; solutions of continuity 109 in 1815, and 199 in 1814.

"On comparing this table with that of last year, we see, that in 1815, putrid, asthenic, and typhous fevers, phlegmasiæ of the mucous and serous membranes, spasmodic and local nervous affections, particular organic lesions and gangrenous inflammations, have been less destructive or less numerous, than in 1814; and that inflammations of the skin, those of the cellular membrane, comatose affections, and general organic lesions, were more frequent or fatal in 1815."—*Journal de Pharmacie, Mai,* 18 6.

---

*Remarks on the Means of discovering the Presence of Arsenic.*—M. Gærtner, of Hanau, "having been charged to examine ham, the use of which had caused the death of several persons, discovered in it the presence of arsenic. After exposing the analytical processes which he employed, M. Gærtner deduces from them the following results:"—"1st. Lime water can only be employed as a re-agent to discover the presence of arsenic, when the suspected liquid contains no marine salt; at least, unless we separate from such liquid the sulphuric acid, magnesia, (invariable constituents of marine salt), and, in general, all substances capable of forming with lime, bodies soluble or insoluble in water. 2dly. Sulphuretted hydrogen gas, recently prepared, (employed at the moment of extrication) is the most delicate of all tests. Its action is much less susceptible of being modified by the presence of other bodies than that of lime water, or even the acidulous hydro-sulphuretted liquor of Hahnemann. 3dly. Yet, as the arsenical sulphuret, separated by this gas from serum and gelatine, is always precipitated with these animal substances, it ought to be wholly deprived of them by sublimation, previously to being subjected to the experiment of the plate of copper: without this, the presence of the poison cannot be rigorously ascertained by the characters of the vapour which it exhales. 4thly. Nitrate of barytes is not decomposed by the acidulous hydro-sulphuretted liquor, but muriate of barytes is. Hence the first merits the preference to the other in an analysis of this nature. 5thly. Ammoniaret of copper is an uncertain test; because, if the arsenical solution contain substances, however small in quantity, capable of altering the effects of this agent, it produces such as are almost inappreciable by the eye. It ought, therefore, to be employed with great circumspection; and can only afford certain results when such substances have been separated from the arsenical solution."—*Journal Universel des Sciences Medicales, Fevrier,* 1816.

---

### *Experiments on Cantharides.*

( Continued from our last Number, page 88. )

3. Two drachms of *sulphuric acid,* diluted with an equal quantity of water, and mixed with one drachm and a half of the *tincture,* assumed an amber colour less transparent, and some shades deeper than No. 1. After twelve hours, a scanty flocculent light brown sediment, partly suspended over the bottom of the glass.

4. Two drachms of *nitric acid* mixed with an equal portion of the *tincture.* Mixture, at first, pale, greenish yellow, transparent. After the lapse of one hour and forty minutes, sudden and violent ebullition with copious extrication of heat and fumes of nitrous gas. After twenty-four hours, transparent, colourless, except a very slight tinge of green. No precipitation; no oily appearance on the surface.

5. Two drachms of *muriatic acid* diluted with as much water, and mixed with one drachm and a half of the *tincture.* Colour, fine greenish-yellow; transparent. Unchanged, and without precipitate, at the end of twelve hours.

6. Twenty grains of *subcarbonate of potash*, dissolved in half an ounce of water: two drachms of the *tincture* poured in. After about an hour, a heavy grey cloud, suspended, a little below the surface: the liquor beneath, of a bright pale amber colour. In a few hours, the cloud slowly and gradually subsided in a convex form. On the following day, nothing to be seen but a scanty, light, brownish-white precipitate; the liquid retaining its former colour and transparency.

7. Twenty grains of *sulphuret of potash*, dissolved in half an ounce of water; a small insoluble portion separated; and two drachms of the *tincture* added to the solution. After a few minutes, an appearance of numerous greyish clots partly deposited, partly suspended, in the fluid, which was seen, in the interstices, of a bright amber colour. On the following morning, the whole precipitated in the form of an abundant, light, yellowish-white powder. Colour of the fluid unchanged.

8. Two drachms of a solution of *prussiate of potash*, poured into an equal quantity of the *tincture*: mixture pale, semi-transparent, whey colour; and soon beginning to deposit a copious greenish-grey sediment. After four hours, the precipitate had assumed a dirty brownish-green colour; and in the fluid above, of a transparent and faint greenish hue, swam numerous minute globules of an apparently oily substance. A few grains of the *solid prussiate*, added to some *tincture*, changed it to a faint green, semi-transparent, whey-like colour, without any evident precipitate, except an undissolved portion of the prussiate.

9. No perceptible change, except a slight degree of turbidity, no precipitation, observed on mixing two drachms of the *tincture* with half an ounce of strong infusions of green and black tea separately. The mixtures remained unaltered at the end of four hours.

I have thus delivered an account of a few simple experiments upon the Tinctura Lyttæ of the London College. How widely different is their result from that obtained by M. Orfila with the French Tincture, the Reader, who will take the trouble of perusing the Review of his first volume in our last Number, must be aware. I pledge myself for the accuracy of the experiments just delineated.

One of the great excellencies of M. Orfila's work is the exposition which he gives of the various chemical phænomena display-ed by poisons in their combinations with different alimentary substances. But the value of this notice is, perhaps, diminished, at least to British practitioners, by his total exclusion of preparations of *Malt* from these researches. I propose, therefore, attempting to remedy this defect; and occasionally to devote a page or two of the Journal to a Description of the Chemical Phænomena exhibited by all the more important Mineral Poisons, in their combinations with the different species of Malt Liquor.

SHIRLEY PALMER.

---

### *To the Editors of the Medico-Chirurgical Journal & Review,*

GENTLEMEN,

WILL ye receive a sort of *critique* on a paragraph or period, in the last Report of the National Vaccine Establishment, addressed to the Secretary of the Home Department, and signed by the Presidents of the Royal Colleges, the Censors of that of Physicians, and the Governors of that of Surgeons.

To Lord Sidmouth it may be news, and being, himself, the son of a physician, he may receive it *con amore;* but, the fact was published in 1803, of which they say,—"that by the ingenuity of Mr. Giraud, of Feversham, means have been devised of preserving the lymph," as they continue to call it, "in a fluid state, by which we have just reason to hope, that it may be found efficient in any climate, and for any space of time."

"My method (says the author) is as follows: I make use of a small glass bulb, with a shank or tube to it, &c. I select a subject with a full pustule, in which I make a small puncture; in a few seconds, a small drop of matter is to be seen on this puncture; I then dip my glass bulb into a cup of boiling water, holding it therein long enough for the expanded air to escape; I then take it out and instantly apply the orifice of the tube to the drop of matter, which will be drawn into the tube as the bulb cools; I then immediately seal the tube, hermetically, by holding the end of it in the flame of a candle.

"To make use of the matter thus preserved, the extreme end of the tube must be broken, and the point of a lancet applied to it; at the same time, by gently approaching the bulb to the flame of a candle, the matter will, by the expansion of the air, be driven out and received on the point of the lancet. Or a puncture may be made on the arm, into which the end of the tube may be inserted, and then the candle gently applied to the bulb.

"Vaccine matter preserved by this method has been inserted, at various periods, from one week to upwards of two months, with complete success. "I am, &c.

*Feversham, April* 10, 1803. "JOHN THOMAS GIRAUD."

A number of these tubes was sent to the Royal Jennerian Society for adoption. The Resident Inoculator declined the trouble attending the use of them. If he had adopted them, the Medical Council of that day would, probably, have obtained his dismissal with disgrace, because of the boiling water, flame of candle, and hermetical sealing employed in the process; for, Doctor Jenner had referred the death of a child, under inoculation for the cow-pox, to the deterioration of the vaccine matter, from its having been held before the fire to dry, when taken by the inoculator.

Now, notwithstanding these great authorities profess to have just reason to hope, as above, I suspect that they cannot have, individually, sufficiently weighed the conjecture which they have thus sanctioned with their signatures.

An eminent apothecary, whose practice occasionally extended to the royal couches in Windsor Castle, called on me, about twelve years ago, for a supply of vaccine ichor. On my presenting to him, some pairs of glasses already charged, he did not make selection of such as by the unevenness of their surfaces afforded intervening space sufficient for collection and continuance of the matter, in a fluid state; but, on the contrary, observing, that he knew not what the consequence might be of a fermentation being set up in this animal substance, which it was ever liable to, while it continued fluid, he preferred the glasses between which the matter was become perfectly quiescent, from its being dry.

Perhaps the fear of the apothecary was founded upon principles more accurate, than is the hope of the two Colleges. Let us not be carried away, then, by any authorities, however eminent. The vaccine matter is, in hot climates, most effectually preserved in its cells or in the interstices of the cellular tissue or texture of the pock, as dried on the arm, in the form of crust or scab, though Dr. Jenner and several other practitioners have entertained the notion, that it never ought to be taken after the formation of the areola. Respectfully,

JOHN WALKER.

*Bond Court, Walbrook, xi, vij,* 1816.

---

*Royal College of Surgeons.—Jacksonian Prize*.—The prize subject, for the year 1817, for the premium of 10*l.* to the author of the best Dissertation on a practical subject in Surgery, is "Deafness, and the Diseases and Injuries of the Organ of Hearing." Candidates must be members of the College, not on the Court of Assistants. The Dissertations must be in English; and the number and importance of the facts will be considered the chief points of excellence in the compositions. Each Dissertation must be distinguished by a motto or device; but no Dissertation, motto, or device, must be in the hand-writing of the author. Every Dissertation must be accompanied by a paper, sealed up (not with the seal of the author), containing the name and residence of the author; and having, on the outside, a motto or device (not in his own hand-writing), corresponding with the motto or device on the Dissertation. Dissertations must be addressed to the Secretary, and delivered at the College before Christmas-day, 1817. The adjudication will be in the month of April, 1818. The Prize Dissertation will be preserved in the library of the College. The unapproved Dissertations, with their correspondent unsealed papers, will be returned, upon authenticated applications. Every unapproved Dissertation, which shall remain three years unclaimed, and its correspondent paper, unopened, will be burnt in the presence of the Jacksonian Committee.—The prize subjects for the present year, 1816, are, "Scrofula" and "Syphilis;" Dissertations upon which must be delivered before Christmas-day next.

The Physico-Medical Society of the University of Erlangen has conferred the honour of its diploma on Dr. Jenner, Sir Humphrey Davy, Professor Brande, and Dr. Burrows.

Dr. P. M. LATHAM and Dr. SOUTHEY will begin their Lectures on the Practice of Physic in the first week of October, at the Middlesex Hospital.

DIED.—In Leicester Square, aged 76, Robert Bland, M. D. —At Enesbury, aged 80, Henry Krohn, M. D. formerly Physician to the London Hospital.—At Bath, Mr. William Vincent, Surgeon, formerly of Sheerness, Kent.

